# AutoTag and AutoSnap: Standardized, semi-automatic capture of regions of interest from whole slide images

**DOI:** 10.1016/j.mex.2015.05.002

**Published:** 2015-05-21

**Authors:** Koen M. Marien, Luc Andries, Stefanie De Schepper, Mark M. Kockx, Guido R.Y. De Meyer

**Affiliations:** a Laboratory of Physiopharmacology, University of Antwerp, Universiteitsplein 1, B-2610 Antwerp, Belgium; b HistoGeneX NV, ZNA Middelheim Campus, Lindendreef 1, B-2020 Antwerp, Belgium

**Keywords:** AutoTag and AutoSnap, Microvessels, Computer-assisted image processing, Selection bias, Automation, Stereology, Whole slide image

## Abstract

Tumor angiogenesis is measured by counting microvessels in tissue sections at high power magnification as a potential prognostic or predictive biomarker. Until now, regions of interest^1^ (ROIs) were selected by manual operations within a tumor by using a systematic uniform random sampling^2^ (SURS) approach. Although SURS is the most reliable sampling method, it implies a high workload. However, SURS can be semi-automated and in this way contribute to the development of a validated quantification method for microvessel counting in the clinical setting. Here, we report a method to use semi-automated SURS for microvessel counting:

• Whole slide imaging with Pannoramic SCAN (3DHISTECH)
• Computer-assisted sampling in Pannoramic Viewer (3DHISTECH) extended by two self-written AutoHotkey applications (AutoTag and AutoSnap)
• The use of digital grids in Photoshop^®^ and Bridge^®^ (Adobe Systems)

This rapid procedure allows traceability essential for high throughput protein analysis of immunohistochemically stained tissue.

## Method details

### Whole slide imaging

**Fig. 1:**
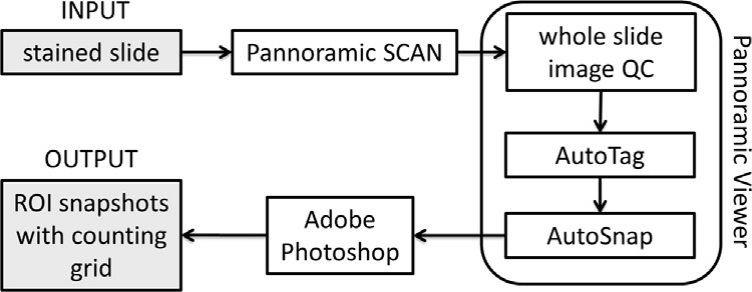
Snapshots of regions of interest (ROIs) combined with a counting grid of a stained slide can be produced semi-automatically and with full traceability. After the stained slide has been scanned in the Pannoramic SCAN (3DHISTECH), it needs to be checked manually for possible image quality issues (out-of-focus regions, incomplete images) in Pannoramic Viewer (3DHISTECH). In the next step, point annotations are semi-automatically created in the whole slide image with AutoTag. Standardized snapshots of the regions of interest (ROIs) can now be automatically created with AutoSnap in Pannoramic Viewer. Finally, a digital grid in Photoshop and Bridge (Adobe Systems) is combined with the snapshots of the ROIs.

**Fig. 2:**
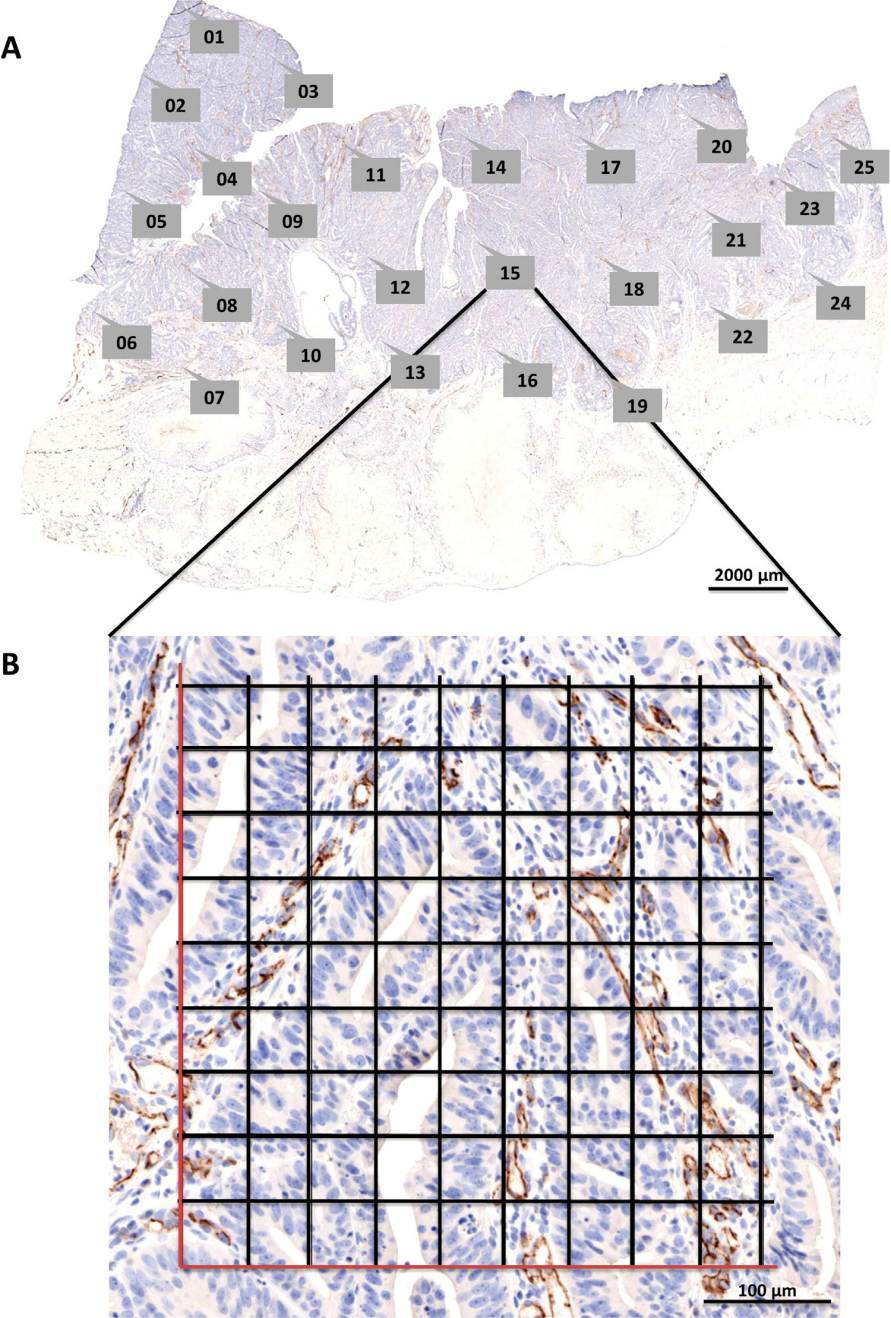
(A) A whole slide image (WSI) of a CD31-stained colorectal carcinoma sample was taken with Pannoramic SCAN. Labeled callout boxes within the WSI display indicate annotations that were semi-automatically created with AutoTag. Subsequently, snapshots for each point annotation were made with AutoSnap. Scale bar = 2000 μm. (B) The resulting snapshot of region of interest #15 is displayed. The digital grid was generated with Adobe Photoshop^®^. Scale bar = 100 μm.

Tumor tissue was stained for CD31 (NCL-CD31-1A10, Leica Biosystems, Diegem, Belgium) on a Ventana Benchmark^®^ XT platform in an accredited laboratory (HistoGeneX NV, Antwerp, Belgium). The stained slides were scanned with the slide scanner Pannoramic SCAN (3DHISTECH) to obtain a whole slide image (Fig. 1). A 20× Plan Apo objective (0.80 NA^3^) and Hitachi (HV-F22CL) 3CCD progressive scan color camera with a resulting image resolution of 0.23 μm/pixel were used. JPEG image encoding with quality factor 80 and an interpolated focus distance of 15 with stitching in the scan options were chosen. For every slide a specific scan profile was configured and holes in the scan area were filled to allow for correct detection of tissue and in-focus images of the tissue. Scanned images were examined to check for image quality and to confirm that the whole tissue section was captured (Fig. 2A).

### Computer assisted sampling

When generating a point annotation in Pannoramic Viewer (build 1.15.4.43061, 3DHISTECH) at a chosen ROI (Fig. 2A), a name for the annotation is requested. To automate repetitive tasks in a Microsoft Windows^®^ environment, the open-source macro-creation and automation software utility AutoHotkey can be used. Semi-automatic naming of point annotations in Pannoramic Viewer during manual annotation was performed with a self-written AutoHotkey script AutoTag running in the background and monitoring the activity on the screen. It names the point annotations with sequentially increasing numbers starting from one (Supplementary Code AutoTag: lines 183–210), allowing the quick generation of point annotations by merely clicking a number of locations in the image.

**Fig. 3:**
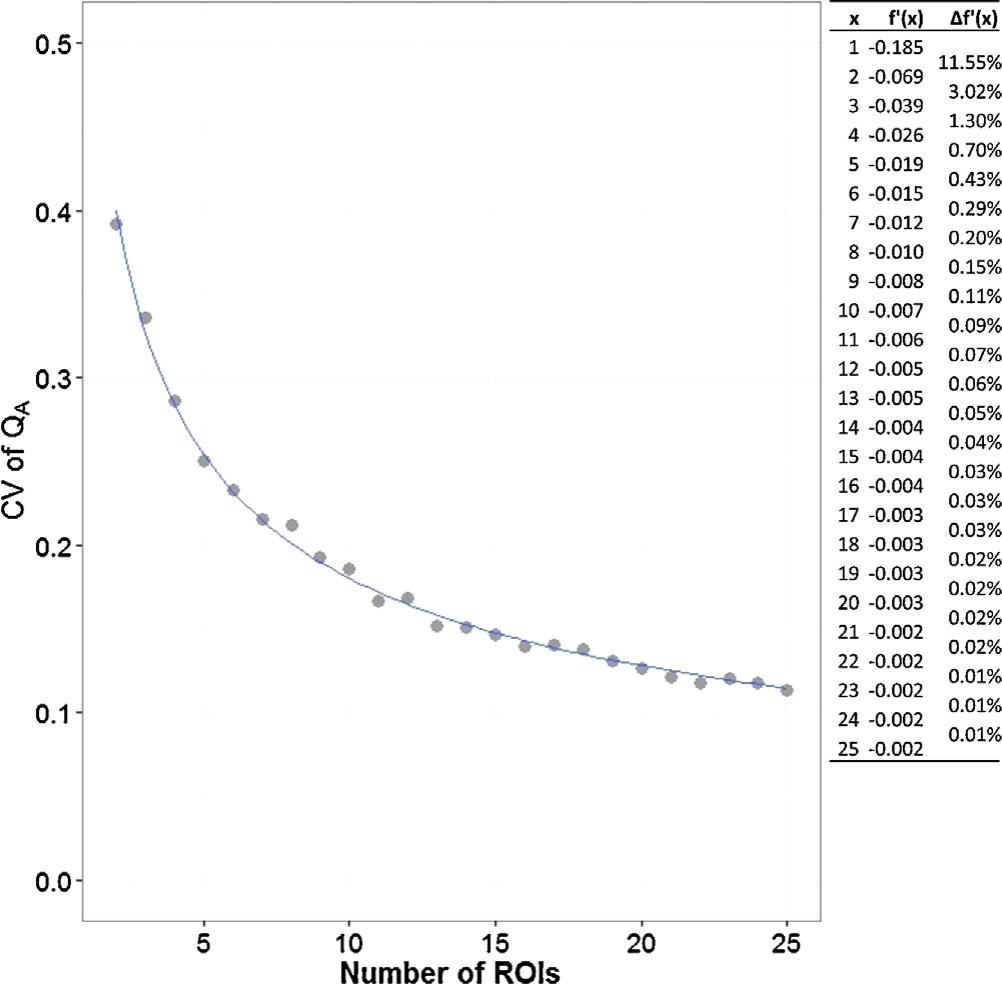
Calculation of the minimum number of regions of interest (ROIs) required for analysis of microvessel density. Twenty-five ROIs were chosen in a CD31-stained colorectal carcinoma slide (see Fig. 2). Random sampling with replacement (i.e. bootstrapping) was carried out 1000 times for the calculation of microvessel densities (*Q_A_*, expressed as number of microvessels per area). The coefficient of variation (CV) was plotted versus the number of ROIs (*f*(*x*) = 0.4446*x*^−0.42^, *R*^2^ = 0.99). The first derivative of this function (*f*′(*x*) = −0.1867*x*^−1.42^) was used to define the minimum number of ROIs required. This value was reached when the difference between two consecutive local derivatives was smaller than 0.50% (in the example shown, the minimum number of ROIs required was six).

The minimum number of regions of interest (ROIs) required for analysis of microvessel density was calculated by using random sampling with replacement (bootstrapping; Fig. 3). Twenty-five ROIs were chosen in a CD31-stained colorectal carcinoma slide (Fig. 2). Microvessel densities (*Q_A_ *= Σ*_i_N_i_*/Σ*_i_V_i_*_,ref_ with an *i* value from 1 to 25 ROIs, expressed as number of microvessels per area, with *N_i_* the number of counted vessels in ROI *i* and *V*_*i*,ref_ the number of grid points hitting tissue in ROI *i*) were calculated 1000 times by resampling from the pool of 25 ROIs. The coefficient of variation (CV) was plotted versus the number of ROIs (Fig. 3). A trend line of the form *f*(*x*) = *ax*^−b^ was added and the first derivative of this function (*f*′(*x*) = −*abx*^−b−1^) was used to define the minimum number of ROIs required. The local derivative must be smaller than 0.50%. After creating these graphs (Fig. 3) for 19 colorectal carcinomas, 22 renal cell carcinomas, 21 glioblastomas, and 21 ovarian carcinomas, we concluded that 10 regions of interest are sufficient for accurate microvessel density measurements.

When all necessary point annotations are created, the magnification is asked by the AutoTag application (Supplementary Code AutoTag: lines 104–142). In general, a 20× magnification is sufficient for the correct identification of microvessels. Finally, AutoTag generates a .txt file that lists the processed samples in column one, the number of semi-automatically created annotations in column two and the magnification in column three (Supplementary Code AutoTag: lines 3–17). This file can be read by a second AutoHotkey application, named AutoSnap, allowing the automatic creation of images of all the ‘AutoTagged’ samples (Fig. 2A). The point annotation will be in the exact center of the snapshot. The created images have a standardized name and are saved at a specified location (Supplementary Code AutoSnap: lines 473–603), allowing batch processing and eliminating the time-consuming process of image creation. Compared to manual image creation by experienced users (19 s; *n* = 832), semi-automatic image generation requires more time on average to construct one image at a given point annotation (66 s; *n* = 272). The process could be speeded up, but is more robust when pauses (sleep functions) are built-in to handle lag due to software processing and network speed. However, due to the automatic nature of AutoSnap no user input is required and, therefore, the process can be run overnight and frees time for other activities. Importantly, the AutoTag and AutoSnap applications semi-automate a sampling method that allows traceability due to the final results being linked to the exact locations in each tissue section.

### Digital grids

To allow unbiased counting of the microvessels, two digital grids with a transparent background (5 by 5 fields and 9 by 9 fields, each 2500 μm^2^, for 40× and 20× magnification, respectively) were created in Adobe Photoshop^®^ (see Supplementary Material). These grids are applied to the images created with AutoSnap in a batch process with Adobe Bridge^®^ (Fig. 2B). A self-written Photoshop^®^ action (see Supplementary Material) automatically pastes the grid as an extra image layer and saves this as a new image.

## Additional information

Tumor angiogenesis is most frequently measured by counting the smallest vessels, known as microvessels, in tumor tissue sections viewed at a high magnification (200×– 400×) [2], [9], [10], [11]. Different immunohistochemistry (IHC) staining assays have been performed to stain blood vessels, including CD31 [7]. The resulting microvessel density score is prognostic for survival in several cancer types [3], [6]. Microvessel counting is performed in a portion of the total tissue area. Usually, one to five ‘hotspots’ with the highest density of microvessels are selected for counting at low magnification [2]. Alternatively, a limited number of regions of interest (ROIs) are identified using a systematic, uniform random sampling (SURS) approach [5]. Microvessels within ROIs are quantified using a rectangular grid in order to obtain the number of vessel profiles per area (*Q_A_*) and the number of grid points overlapping with vessels per area (*A_A_*) [4]. When the SURS sampling method was compared with other techniques, such as hotspot in breast cancer samples, the variation in the stereological estimate *Q_A_* was highly associated with biological differences between the samples (78%) and a smaller contribution from inherent methodological factors (17%) or observers (5%) [5]. Therefore, it is the most reliable method to identify variation in microvessel density among patients, but it results in a high workload. Recently, a web-based tool was written to create an equidistant set of ROIs within a given region [12]. This tool automates the selection process, but as coordinates of the ROIs are calculated automatically in given region, quality control must be performed to assess suitability of the coordinates. Depending on the analysis this can be necrosis, viable tissue, or a sufficiently high number of cells of interest. It is also not possible to create images at the coordinates and no grids are overlaid. Microvessel counting can be performed manually on the images created by this process, or can be used for automatic image analysis when the digital grid is discarded [1], [8].

## Footnotes

3 NA: numerical aperture.
