# Supplementary material for: AutoTag and AutoSnap: Standardized, semi-automatic capture of regions of interest from whole slide images
Source: MethodsX. 2015 May 21;2:272–7. doi: 10.1016/j.mex.2015.05.002 (PMC4487922; doi:10.1016/j.mex.2015.05.002)
Supplement: Supplementary file 1 [file mmc1.docx]

## Supplementary Code

### AutoTag

#MaxThreadsPerHotkey 20

; Create log file

FileSelectFolder, LocationLog,,,Where do you want to save the log file?

LocationLog2 = %LocationLog%\log.txt

If FileExist(LocationLog2)

{

Return

} else {

FileAppend, User Start End Action, %LocationLog2%

if (ErrorLevel <> 0)

{

MsgBox, 48, Error, There is a problem with the creation of the log file.`n`nThe application will now close.

Return

}

}

; Index the Mirax images

FileSelectFolder, ImagesPath,,,Where are the images located? (Provide a folder with only the images you want to annotate)

ImageName:

ImagesList =

Loop, %ImagesPath%\*, 0, 0 ; _1.mrxs is also indexed

{

FoundPos := RegExMatch(A_LoopFileName, "i)\.mrxs$")

if FoundPos = 0

{

MsgBox, 21, Error, The folder you selected does not only contain images (%A_LoopFileName%). Please adjust and retry.

IfMsgBox, Retry

GoTo, ImageName

IfMsgBox, Cancel

ExitApp

}

ImagesList = %ImagesList%%A_LoopFileName%`n

NumberOfImages = %A_Index%

}

If (ImagesList = "")

{

MsgBox, 48, Error, The folder you selected (%ImagesPath%\) does not contain any images. Please try again.`n`nThe application will now close.

ExitApp

}

; Retrieve Windows user name

WinUser = %A_UserName%

; Remember start time

FormatTime, StartTime,, d/MM/yy HH:mm

; Check if there already annotations have been made and create folders and file if not

FileSelectFolder, LocationAnnotations,,,Where do you want to save the annotations file?

LocationAnnotations2 = %LocationAnnotations%\annotations.txt

Samples = 0

Next = 0

If FileExist(LocationAnnotations2)

{

FileRead, Text, %LocationAnnotations2%

Loop, Parse, Text, `n, `r

Next := A_Index ; Header line included

Samples := Next - 1 ; Do not count header line

If Samples = %NumberOfImages%

{

FormatTime, EndTime,, d/MM/yy HH:mm

FileAppend, `n%WinUser% %StartTime% %EndTime% AutoTag: already annotated, %LocationLog2%

MsgBox, 64, Info, All images have been already annotated.`n`nThe application will now close.

ExitApp

}

If Samples > %NumberOfImages%

{

MsgBox, 32, Error, Are you sure you entered the correct information?`n(%Samples% have been already annotated and %NumberOfImages% images are present.)`n`nPlease correct and run the program again.

ExitApp

} else {

MsgBox, 64, Info, There have been made annotations already for %Samples% of the %NumberOfImages% samples. You will continue with sample %Next% of the request.

}

} else {

FileAppend, SampleID NumberOfAnnotations Magnification, %LocationAnnotations2%

if (ErrorLevel <> 0)

{

MsgBox, 48, Error, There is a problem with the creation of the annotations text-file.`n`nThe application will now close.

ExitApp

}

}

; Create annotations and save information for each sample

MsgBox, 64, Info, Press Windows key + 'T' to start recording the annotations for a new sample. Press these keys again to stop recording`, and save the information. `nPress Escape to stop the program.

Index = %Samples%

#t::

#MaxThreadsPerHotkey 1

if KeepWinTRunning ; This means an underlying thread is already running the loop below.

{

SetTimer,CheckWin,Off

KeepWinTRunning := false ; Signal that thread's loop to stop.

MagSelection:

Gui, 4:Add, Text,, Which magnification have you been using?

Gui, 4:Add, Radio, VFit, Fit

Gui, 4:Add, Radio, VTwenty Checked1, 20x

Gui, 4:Add, Radio, VFourty, 40x

Gui, 4:Add, Radio, VFifty, 50x

Gui, 4:Add, Button, Default, OK

Gui, 4:Show, W225 H125, Magnification

Return

4ButtonOK:

Gui, 4:Submit

if (Fit = 1){

Magn = Fi

} else {

if (Twenty = 1){

Magn := 20

} else {

if (Fourty = 1){

Magn := 40

} else {

if (Fifty = 1){

Magn := 50

} else {

MsgBox, 21, Error, No magnification was selected.

IfMsgBox, Retry

GoTo, MagSelection

IfMsgBox, Cancel

ExitApp

}

}

}

}

Gui, 4:Destroy

FileAppend, `n%ID% %Counter% %Magn%, %LocationAnnotations2%

Gui, 3:Destroy

Return ; End this thread so that the one underneath will resume and see the change made by the line above.

} ; Otherwise:

KeepWinTRunning := true

++Index

If Index > %NumberOfImages%

{

FormatTime, EndTime,, d/MM/yy HH:mm

FileAppend, `n%WinUser% %StartTime% %EndTime% AutoTag: Finish, %LocationLog2%

MsgBox, 64, Info, All images have been annotated!`n`nThe application will now close.

ExitApp

}

Current =

Loop, Parse, ImagesList, `n

{

if Index = %A_Index%

{

StringTrimRight, Current, A_LoopField, 5 ; remove '.mrxs'

ID = %Current%

}

}

;TrayTip, Info, Autotag activated!,,1

Gui, 3:+AlwaysOnTop

Gui, 3:Font, S10 , Verdana

Gui, 3:Add, Text,, You need to make annotations for the following image:

Gui, 3:Add, Text, CBlue, %ID%

Gui, 3:Color, Yellow

Gui, 3:Show, X0 Y0 AutoSize, Info

SetTitleMatchMode 2 ;A window's title can contain WinTitle anywhere inside it to be a match.

Check:

IfWinNotExist, %ID%

{

MsgBox, 32, Error, Currently you are not viewing the image of %ID%. Please open it, maximize it and click OK.

Goto, Check

}

SetTimer,CheckWin

Counter = 0

Loop

{

SetTitleMatchMode 1 ;A window's title must start with the specified WinTitle to be a match

KeyWait, LButton, D

if not KeepWinTRunning ; The user signaled the loop to stop by pressing Win-T again.

break ; Break out of this loop.

WinWaitActive, Create ;Wait until annotation window 'Create ...' exists and is active

if ErrorLevel = 1

MsgBox, 32, Error, WinWaitActive timed out.

++Counter

WinActivate

Sleep 50

if (Counter < 10)

{

Send +{End}

Sleep 50

Send 0%Counter%

} else {

Send +{End}

Sleep 50

Send %Counter%

}

Sleep 50

Send {Enter}

Sleep 50

}

KeepWinTRunning := false ; Reset in preparation for the next press of this hotkey.

Return

CheckWin:

SetTitleMatchMode 2

IfWinNotExist, %ID%

{

MsgBox, 32, Error, Currently you are not viewing the image of %ID%. Please open it, maximize it and click OK.

Goto, CheckWin

}

SetTitleMatchMode 1

Return

Esc::

FormatTime, EndTime,, d/MM/yy HH:mm

FileAppend, `n%WinUser% %StartTime% %EndTime% AutoTag: Escape, %LocationLog2%

ExitApp ;Escape key will exit... place this at the bottom of the script

### AutoSnap

; Information where in the register hidden extensions status is stored

GroupAdd ExplorerWindows, ahk_class ExploreWClass|CabinetWClass|Progman

SubKey := "Software\Microsoft\Windows\CurrentVersion\Explorer\Advanced"

MsgBox, 64, AutoSnap, Press Windows key + 'I', to start snapshotting.

#i::

; Status show/hide of extensions for known file types

Hide =

Hide := GetRegValue("HideFileExt")

if (Hide = 1) {

MsgBox, 48, Hidden Extensions, File extensions are not shown and this is needed for the correct working of the script. This option will now be changed. Please restart Pannoramic Viewer.

SetRegValue("HideFileExt", 0)

gosub UpdateWindows

}

; Project number and path:

FileSelectFolder, LocationLog,,,Where do you want to save the log file?

; Create log file

LocationLog = %LocationLog%\log.txt

if FileExist(LocationLog) {

} else {

FileAppend, User Start End Action, %LocationLog%

if (ErrorLevel <> 0)

{

MsgBox, 48, Error, There is a problem with the creation of the log file.`n`nThe application will now close.

ExitApp

}

}

; Index the Mirax images

FileSelectFolder, ImagesPath,,,Where are the images located?

ImagesList =

Loop, %ImagesPath%\*, 0, 0 ; _1.mrxs is also indexed

{

ImagesList = %ImagesList%%A_LoopFileName%`n

NumberOfImages = %A_Index%

}

; Retrieve Windows user name

WinUser = %A_UserName%

; Remember start time

FormatTime, StartTime,, d/MM/yy HH:mm

; Location of annotations text file

FileSelectFolder, LocationAnnotations,,,Where is the annotations file located?

LocationAnnotations = %LocationAnnotations%\annotations.txt

; Location of snapshots

FileSelectFolder, LocationSnaps,,,Where do the snapshots have to be saved?

MsgBox, Don't forget to turn 'Show Scalebar' on in 'Capture Image'.`n`nScroll down in the folder tree until all images are visible.`n`nActivate the Pannoramic Viewer window and place your mouse cursor on the folder in Pannoramic Viewer`, but do NOT select it.`n`nThen Press Windows key + space`, to start the automatic snapshot procedure.`n`nDuring the automatic snapshotting, keyboard and mouse will not work. You can unlock this security measure by pressing Ctrl+Alt+Del(ete) (multiple times if needed), logging into Windows and pressing Esc.

Return

#space::

; Clear clipboard

clipboard =

; Block user input (mouse and keyboard)

KeyWait, LWin ;Wait for release of LWin, RWin and Space key, otherwise BlockInput doesn't work well

KeyWait, RWin

KeyWait, Space

BlockInput, On

SetTimer,LockStation ; Ctrl-Alt-Del stops the blocked input and asks user for password

; Record mouse coordinates of image folder in Pannoramic Viewer

MouseGetPos, xpos, ypos

SetKeyDelay, 100

SetMouseDelay, 100

; Read annotations.txt

FileRead, Text, %LocationAnnotations%

Loop, Parse, Text, `n, `r

Samples := A_Index - 1 ;Do not count header line

; Show annotations.txt content

;MsgBox, 64, annotations.txt, The annotations.txt file read in, contains the following information: `n`n%Text%

; Check annotations.txt

if NumberOfImages <> %Samples%

{

BlockInput, Off

SetTimer,LockStation,Off

FormatTime, EndTime,, d/MM/yy HH:mm

FileAppend, `n%WinUser% %StartTime% %EndTime% AutoSnap: different number of annotations and images, %LocationLog%

MsgBox, The number of samples in 'annotations.txt' (%Samples%) does not match the number of images (%NumberOfImages%).`n`nPlease check.

Return

}

; Check magnification and create folders, remember their location

CounterB = 0

while CounterB < Samples {

++CounterB

FileReadLine, line, %LocationAnnotations%, % CounterB + 1 ;%+1 because of header line in annotations.txt

Magn := SubStr(line, -1, 2)

if (Magn = 20) {

FileCreateDir, %LocationSnaps%\20x ; Create the 20x folder

LocationSnaps20 = %LocationSnaps%\20x\

Continue

} else {

if (Magn = 40) {

FileCreateDir, %LocationSnaps%\40x ; Create the 40x folder

LocationSnaps40 = %LocationSnaps%\40x\

Continue

} else {

if (Magn = "Fi") {

FileCreateDir, %LocationSnaps%\Fit ; Create the fit folder

LocationSnapsFi = %LocationSnaps%\Fit\

Continue

} else {

if (Magn = 50) {

FileCreateDir, %LocationSnaps%\50x ; Create the 50x folder

LocationSnaps50 = %LocationSnaps%\50x\

Continue

} else {

BlockInput, Off

SetTimer,LockStation,Off

FormatTime, EndTime,, d/MM/yy HH:mm

FileAppend, `n%WinUser% %StartTime% %EndTime% AutoSnap: wrong magnification in annotations file, %LocationLog%

MsgBox, Magnification does not match 'Fi', '20', '40' or '50' (%Magn% has been read) in line %CounterB% in %LocationAnnotations%.

Return

}

}

}

}

}

; Check if there have already snapshots been made and which the last one is (ID + annotation)

Test =

LastSnapFi =

TagFi = 0

Current =

Current2 =

RowFi = 0

OrigFi = 0

FoundPosFi = 0

IfExist, %LocationSnapsFi%*.bmp ; If there are annotations for Fit

{

Loop, %LocationSnapsFi%*.bmp, 0, 0 ; _1_XX.bmp are also indexed

{

LastSnapFi = %A_LoopFileName% ; What is the last snapshot?

}

TagFi := SubStr(LastSnapFi, -5, 2) ; Remember the number of the annotation

TagFi := RegExReplace(TagFi, "^0") ; Remove any leading zeros

StringTrimRight, LastSnapFi, LastSnapFi, 7 ; remove _XX.bmp

FileRead, Text, %LocationAnnotations% ; Which line contains the SampleID(_X) in annotations.txt?

Loop, Parse, Text, `n

{

FoundPosFi := RegExMatch(A_LoopField,"\t")

StringLeft, Current, A_LoopField, FoundPosFi - 1 ; Retrieve SampleID(_X) in annotations.txt

StringMid, Current2, A_LoopField, FoundPosFi +1, 2

Current2 := RegExReplace(Current2, "^0") ; Remove any leading zeros

if Current = %LastSnapFi%

{

OrigFi := A_Index

if Current2 > %TagFi% ; If number of total annotations (annotations.txt) > annotation number of snapshot

{

RowFi := A_Index - 2 ; Subtract the header line and start with this SampleID

} else {

RowFi := A_Index - 1 ; Subtract the header line and skip this SampleID

TagFi = 0 ; Reset CounterT to zero

}

}

}

}

Test =

LastSnap20 =

Tag20 = 0

Current =

Current2 =

Row20 = 0

Orig20 = 0

FoundPos20 = 0

IfExist, %LocationSnaps20%*.bmp ; If there are annotations for 20x

{

Loop, %LocationSnaps20%*.bmp, 0, 0 ; _1_XX.bmp are also indexed

{

LastSnap20 = %A_LoopFileName% ; What is the last snapshot?

}

Tag20 := SubStr(LastSnap20, -5, 2) ; Remember the number of the annotation

Tag20 := RegExReplace(Tag20, "^0") ; Remove any leading zeros

StringTrimRight, LastSnap20, LastSnap20, 7 ; remove _XX.bmp

FileRead, Text, %LocationAnnotations% ; Which line contains the SampleID(_X) in annotations.txt?

Loop, Parse, Text, `n

{

FoundPos20 := RegExMatch(A_LoopField,"\t")

StringLeft, Current, A_LoopField, FoundPos20 - 1

StringMid, Current2, A_LoopField, FoundPos20 + 1, 2

Current2 := RegExReplace(Current2, "^0") ; Remove any leading zeros

if Current = %LastSnap20%

{

Orig20 := A_Index

if Current2 > %Tag20% ; If number of total annotations (annotations.txt) > annotation number of snapshot

{

Row20 := A_Index - 2 ; Subtract the header line and start with this SampleID

} else {

Row20 := A_Index - 1 ; Subtract the header line and skip this SampleID

Tag20 = 0 ; Reset CounterT to zero

}

}

}

}

Test =

LastSnap40 =

Tag40 = 0

Current =

Current2 =

Row40 = 0

Orig40 = 0

FoundPos40 = 0

IfExist, %LocationSnaps40%*.bmp ; If there are annotations for 40x

{

Loop, %LocationSnaps40%*.bmp, 0, 0 ; _1_XX.bmp are also indexed

{

LastSnap40 = %A_LoopFileName% ; What is the last snapshot?

}

Tag40 := SubStr(LastSnap40, -5, 2) ; Remember the number of the annotation

Tag40 := RegExReplace(Tag40, "^0") ; Remove any leading zeros

StringTrimRight, LastSnap40, LastSnap40, 7 ; remove _XX.bmp

FileRead, Text, %LocationAnnotations% ; Which line contains the SampleID(_X) in annotations.txt?

Loop, Parse, Text, `n

{

FoundPos40 := RegExMatch(A_LoopField,"\t")

StringLeft, Current, A_LoopField, FoundPos40 - 1

StringMid, Current2, A_LoopField, FoundPos40 + 1, 2

Current2 := RegExReplace(Current2, "^0") ; Remove any leading zeros

if Current = %LastSnap40% ; Retrieve number of annotations for that SampleID (no leading zeros present, autotrim of spaces)

{

Orig40 := A_Index

if Current2 > %Tag40%

{

Row40 := A_Index - 2 ; Subtract the header line and start with this SampleID

} else {

Row40 := A_Index - 1 ; Subtract the header line and skip this SampleID

Tag40 = 0 ; Reset CounterT to zero

}

}

}

}

Test =

LastSnap50 =

Tag50 = 0

Current =

Current2 =

Row50 = 0

Orig50 = 0

FoundPos50 = 0

IfExist, %LocationSnaps50%*.bmp ; If there are annotations for 50x

{

Loop, %LocationSnaps50%*.bmp, 0, 0 ; _1_XX.bmp are also indexed

{

LastSnap50 = %A_LoopFileName% ; What is the last snapshot?

}

Tag50 := SubStr(LastSnap50, -5, 2) ; Remember the number of the annotation

Tag50 := RegExReplace(Tag50, "^0") ; Remove any leading zeros

StringTrimRight, LastSnap50, LastSnap50, 7 ; remove _XX.bmp

FileRead, Text, %LocationAnnotations% ; Which line contains the SampleID(_X) in annotations.txt?

Loop, Parse, Text, `n

{

FoundPos50 := RegExMatch(A_LoopField,"\t")

StringLeft, Current, A_LoopField, FoundPos50 - 1

StringMid, Current2, A_LoopField, FoundPos50 + 1, 2

Current2 := RegExReplace(Current2, "^0") ; Remove any leading zeros

if Current = %LastSnap50% ; Retrieve number of annotations for that SampleID (no leading zeros present, autotrim of spaces)

{

Orig50 := A_Index

if Current2 > %Tag50%

{

Row50 := A_Index - 2 ; Subtract the header line and start with this SampleID

} else {

Row50 := A_Index - 1 ; Subtract the header line and skip this SampleID

Tag50 = 0 ; Reset CounterT to zero

}

}

}

}

CounterS = 0

If RowFi > Row20 ; Which snapshot is the latest in annotations.txt, a Fit, 20x, 40x or a 50x?

{

CounterS = %RowFi%

} else {

If Row20 > %Row40%

{

CounterS = %Row20%

} else {

If Row40 > %Row50%

{

CounterS = %Row40%

} else {

CounterS = %Row40%

}

}

}

; Start big loop

CounterP = 0 ; Count how much time the loop has run independent of number of samples

while CounterS < Samples {

; Read number of annotations and magnification

++CounterS

++CounterP

FileReadLine, line, %LocationAnnotations%, % CounterS + 1 ;%+1 because of header line in annotations.txt

Magn := SubStr(line, -1, 2)

FoundPos := RegExMatch(line,"\t")

ID := SubStr(line, 1, FoundPos - 1)

NumberTags := SubStr(line, FoundPos + 1, 2)

CounterT = 0

If CounterP = 1

{

If OrigFi > %Orig20% ; Which snapshot is the latest in annotations.txt, a Fit, 20x, 40x or a 50x?

{

CounterT = %TagFi%

} else {

If Orig20 > %Orig40%

{

CounterT = %Tag20%

} else {

If Orig40 > %Orig50%

{

CounterT = %Tag40%

} else {

CounterT = %Orig50%

}

}

}

}

WinActivate, Pannoramic

Sleep 1000

; When Pannoramic Viewer for the first time is being opened, Tree View and Preview View are on, so they are not allowed to be closed

if CounterP > 1 ; The Tree View needs to be opened again the second iteration so the second image can be opened via the mouse

{

Send ^t

Sleep 500

Send ^p

Sleep 500

}

if NumberTags = 0

{

Send ^t

Sleep 500

Send ^p

Sleep 500

Continue

}

; Open image

MouseMove %xpos%, %ypos%

Sleep 1000

Click ; You need to ask the user to NOT select the folder

Sleep 1000

Send {Down %CounterS%}

Sleep 1000

Send {Enter}

Sleep 10000

Send !{-} ; shortcut for system menu

Sleep 2000

Send {Down 4} ; go to maximize

Sleep 2000

Send {Enter}

Sleep 12000

if (Magn = "Fi") { ; Fi = F2; 20x = F7; 40x = F8

Send {F2}

} else {

if (Magn = 20) {

Send {F7}

} else {

if (Magn = 40){

Send {F8}

} else {

if (Magn = 50){

Send {Tab 4}

Sleep 500

Send {BS 5}

Sleep 500

Send 50

Sleep 500

Send {Enter}

}

}

}

}

Sleep 10000

SetTitleMatchMode 2 ;A window's title can contain WinTitle anywhere inside it to be a match.

Check:

IfWinNotExist, %ID%

{

BlockInput, Off

SetTimer,LockStation,Off

WinGetTitle, Title, A

FormatTime, EndTime,, d/MM/yy HH:mm

FileAppend, `n%WinUser% %StartTime% %EndTime% AutoSnap: wrong image open, %LocationLog%

MsgBox, The image that is open (%Title%) does not correspond with the sample currently being used in annotations.txt (%ID%). Please correct the order in the annotations.txt file to match the order of the images in Pannoramic Viewer.

Return

}

WinActivate, Pannoramic

; Close Tree View and Preview View to allow use on different PCs with different sizes of Tree View and Preview View

Send ^t

Sleep 500

Send ^p

Sleep 500

WinActivate, Pannoramic

while CounterT < NumberTags {

; Start small loop

Sleep 2000

MouseMove 215, 90 ;Go to Manage/annotate locations

Click

Click

Sleep 2000

WinActivate, Manage

MouseMove 59, 57 ;Go to first annotation

Click

Send {Down %CounterT%}

++CounterT

MouseMove 52, 465 ;Go to Locate

Click

Sleep 10000

WinActivate, Pannoramic

MouseMove 275, 90 ;Go to Capture image

Click

Click

Sleep 4000

WinActivate, Image

Sleep 4000

Send {tab 10} ;Go to Save (old pann viewer: 9 <> new pann viewer: 10)

Sleep 2000

Send {Enter}

Sleep 3000

WinActivate, Save

Sleep 3000

if (Magn = "Fi") {

Send {Home}

Sleep 3000

if (CounterT > 9) {

clipboard = %LocationSnapsFi%

ClipWait

Send ^v

Sleep 3000

Send {End}

Sleep 3000

Send {BS 4} ; Instellingen Windows Mapopties hide extensions???

Sleep 3000

clipboard = _%CounterT%

ClipWait

Send ^v

} else {

clipboard = %LocationSnapsFi%

ClipWait

Send ^v

Sleep 3000

Send {End}

Sleep 3000

Send {BS 4}

Sleep 3000

clipboard = _0%CounterT%

ClipWait

Send ^v

}

} else {

if (Magn = 20) {

Send {Home}

Sleep 3000

if (CounterT > 9) {

clipboard = %LocationSnaps20%

ClipWait

Send ^v

Sleep 3000

Send {End}

Sleep 3000

Send {BS 4} ; Instellingen Windows Mapopties hide extensions???

Sleep 3000

clipboard = _%CounterT%

ClipWait

Send ^v

} else {

clipboard = %LocationSnaps20%

ClipWait

Send ^v

Sleep 3000

Send {End}

Sleep 3000

Send {BS 4}

Sleep 3000

clipboard = _0%CounterT%

ClipWait

Send ^v

}

} else {

if (Magn = 40) {

Send {Home}

Sleep 3000

if (CounterT > 9) {

clipboard = %LocationSnaps40%

ClipWait

Send ^v

Sleep 3000

Send {End}

Sleep 3000

Send {BS 4}

Sleep 3000

clipboard = _%CounterT%

ClipWait

Send ^v

} else {

clipboard = %LocationSnaps40%

ClipWait

Send ^v

Sleep 3000

Send {End}

Sleep 3000

Send {BS 4}

Sleep 3000

clipboard = _0%CounterT%

ClipWait

Send ^v

}

} else {

if (Magn = 50) {

Send {Home}

Sleep 3000

if (CounterT > 9) {

clipboard = %LocationSnaps50%

ClipWait

Send ^v

Sleep 3000

Send {End}

Sleep 3000

Send {BS 4}

Sleep 3000

clipboard = _%CounterT%

ClipWait

Send ^v

} else {

clipboard = %LocationSnaps50%

ClipWait

Send ^v

Sleep 3000

Send {End}

Sleep 3000

Send {BS 4}

Sleep 3000

clipboard = _0%CounterT%

ClipWait

Send ^v

}

}

}

}

}

Sleep 3000

WinActivate, Save

Sleep 3000

Send {Enter}

Sleep 3000

IfWinExist, Confirm Save As

{

WinActivate

Send {tab}

Sleep 2000

Send {Enter}

Sleep 2000

}

WinActivate, Image

MouseMove 1547, 968 ; Go to Close

Click

}

Sleep 1000

Send ^{F4} ; shortcut for Close

Sleep 1000

}

Send ^t

Sleep 500

Send ^p

Sleep 500

; Check number of snapshots taken against number in annotations.txt

; Read annotations.txt

FileRead, Text, %LocationAnnotations%

Loop, Parse, Text, `n, `r

counter = 0

NumberTags = 0

NumberAnnFi = 0

NumberAnn20 = 0

NumberAnn40 = 0

NumberAnn50 = 0

while counter < Samples {

++counter

FileReadLine, line, %LocationAnnotations%, % counter + 1 ;% For better reading in Notepad++, +1 because of header line in annotations.txt

Magn := SubStr(line, -1, 2)

FoundPos := RegExMatch(line,"\t")

ID := SubStr(line, 1, FoundPos - 1)

NumberTags := SubStr(line, FoundPos + 1, 2)

if (Magn = "Fi") {

NumberAnnFi := NumberAnnFi + NumberTags

} else {

if (Magn = 20) {

NumberAnn20 := NumberAnn20 + NumberTags

} else {

if (Magn = 40) {

NumberAnn40 := NumberAnn40 + NumberTags

} else {

if (Magn = 50) {

NumberAnn50 := NumberAnn50 + NumberTags

}

}

}

}

}

; Count number of Fit snapshots

NumberSnapsFi = 0

IfExist, %LocationSnapsFi%*.bmp ; If there are annotations for Fit

{

Loop, %LocationSnapsFi%*.bmp, 0, 0 ; _1_XX.bmp are also indexed

{

NumberSnapsFi = %A_Index% ; How many snapshots have been taken?

}

}

; Count number of 20x snapshots

NumberSnaps20 = 0

IfExist, %LocationSnaps20%*.bmp ; If there are annotations for 20x

{

Loop, %LocationSnaps20%*.bmp, 0, 0 ; _1_XX.bmp are also indexed

{

NumberSnaps20 = %A_Index% ; How many snapshots have been taken?

}

}

; Count number of 40x snapshots

NumberSnaps40 = 0

IfExist, %LocationSnaps40%*.bmp ; If there are annotations for 40x

{

Loop, %LocationSnaps40%*.bmp, 0, 0 ; _1_XX.bmp are also indexed

{

NumberSnaps40 = %A_Index% ; How many snapshots have been taken?

}

}

; Count number of 50x snapshots

NumberSnaps50 = 0

IfExist, %LocationSnaps50%*.bmp ; If there are annotations for 50x

{

Loop, %LocationSnaps50%*.bmp, 0, 0 ; _1_XX.bmp are also indexed

{

NumberSnaps50 = %A_Index% ; How many snapshots have been taken?

}

}

BlockInput, Off

SetTimer,LockStation,Off

FormatTime, EndTime,, d/MM/yy HH:mm

FileAppend, `n%WinUser% %StartTime% %EndTime% AutoSnap: finish, %LocationLog%

MsgBox, Your snapshots have been taken! `n`n * Fit: %NumberSnapsFi% created - %NumberAnnFi% requested `n * 20x: %NumberSnaps20% created - %NumberAnn20% requested `n * 40x: %NumberSnaps40% created - %NumberAnn40% requested `n * 50x: %NumberSnaps50% created - %NumberAnn50% requested `n`nPlease check if these numbers don't match.

ExitApp

Return

LockStation:

If !DllCall("GetForegroundWindow"){

DllCall("LockWorkStation")

while !DllCall("GetForegroundWindow")

Sleep, 1000

}

Return

; Send a "Refresh" message to all of the Explorer windows including the Desktop

UpdateWindows:

Code := InStr("WIN_XP, WIN_2000", A_OSVERSION) ? 28931 : 41504

WinGet WindowList, List, ahk_Group ExplorerWindows

Loop %WindowList%

PostMessage 0x111, %Code%, , , % "ahk_id" WindowList%A_Index%

Return

GetRegValue(ValueName) {

global SubKey

RegRead Value, HKCU, %SubKey%, %ValueName%

return Value

}

SetRegValue(ValueName, Value) {

global SubKey

RegWrite REG_DWORD, HKCU, %SubKey%, %ValueName%, %Value%

}

Esc::

FormatTime, EndTime,, d/MM/yy HH:mm

FileAppend, `n%WinUser% %StartTime% %EndTime% AutoSnap: escape, %LocationLog%

ExitApp ;Escape key will exit... place this at the bottom of the script
